# The Effect of Self-Care Compact Disk-Based Instruction Program on Physical Performance and Quality of Life of Patients with Burn At-Dismissal

**DOI:** 10.29252/wjps.8.1.25.

**Published:** 2019-01

**Authors:** Leila Mamashli, Fatemeh Mohaddes Ardebili, Mehri Bozorgnejad, Tahereh Najafi Ghezeljeh, Farzad Manafi

**Affiliations:** 1 Islamic Azad University, Ali Abad Katoul Branch, Ali Abad Katoul, Iran;; 2 Department of Medical-Surgical Nursing, Faculty of Nursing and Midwifery, Iran University of Medical Sciences, Tehran, Iran;; 3 Department of Critical Care Nursing, School of Nursing and Midwifery, Iran University of Medical Sciences, Tehran, Iran;; 4 Burn Research Center, School of Medicine, Iran University of Medical Sciences, Tehran, Iran

**Keywords:** Self-care, Burn, Physical performance, Quality of life, Iran

## Abstract

**BACKGROUND:**

Burn injuries still negatively influence the various aspects of life like physical performance and quality of life. This study was conducted to investigate at-dismissal self-care compact disk-based instruction program on the physical performance life quality of the patients with burns.

**METHODS:**

One-hundred burn patients in Shahid Motahhari Burn Center, Tehran, Iran were randomly assigned to two equal groups of intervention (n=50) and control (n=50). The latter received only routine dismissal self-care program and the former an instruction compact disc plus the routine self-care program at dismissal. The demographic information and burn patients’ quality of life questionnaires were completed before and at months three and six after the intervention self-report program.

**RESULTS:**

The physical performance of the intervention and control groups before intervention was 1.61±0.71 and 1.45±0.47, respectively (*p*=0.41). The physical performance of the intervention group was higher than the control group at three and six months after the intervention (*p*<0.001).

**CONCLUSION:**

At-dismissal self-care compact disk-based instruction program can increase physical performance and quality of life in patients with burns. Therefore, the burn patients can be instructed based on self-care compact disk-based instruction program as an easy, available and less-costly method to take part in more satisfied treatment.

## INTRODUCTION

Burn is one of the most detrimental damages that an individual may experience during his or her life.^1^ Burn is a global crisis regarding general hygiene in global level and severely harms the life and health of an individual and is considered as the fourth most prevalent injury worldwide.^2^ Annually, about 2.4 million burns occur around the globe and 650000 cases of which need treatment, 75000 cases are hospitalized and between 8 and 12 thousand cases are considered as superficial burns.^3^ An epidemiological study in 2017 in ten countries (Australia, Bulgaria, China, Czech Republic, Finland, Iran, Israel, Netherlands, Oman and Britain) shows that the burn prevalence rate is high in these countries.^4^ The burn statistics of Iran, with its 75-million population, are indicative of an annual of 150 thousand cases of burns out of which between 25 and 30 thousand are hospitalized which is reflective of the high rate of burns in Iran.^5^

Burn injuries are the major cause of mortality and disability in the world. In US, burns is the primary cause of death enrolling 3240 individuals annually and with 40000 hospitalizations.^3^ Corresponding to the statistics issued by the country’s forensic medicine organization for 2018, 379 individuals have lost their lives during the first trimester as a result of burns, among them, 213 burn cases were male and 166 were female and the highest number of the burn victims was respectively found in Khuzestan Province (n=51), Fars (n=42), and Tehran (n=37).^6^ Besides death, injuries resulting from burns are undoubtedly amongst the most severe forms of trauma bringing about many disabilities and long-term or chronic symptoms, increase in hospital costs and recurrent hospitalization and physical damage.^7^^,^^8^ It was shown that burns are amongst the accidents imposing numerous physical changes on the victims, including physical difficulties, body organ deformations, scars, wrinkling, skin color changes, protein anabolism and catabolism imbalance, destruction of the musculoskeletal mass and, eventually, cell death.^9^

It was demonstrated that the symptoms pertinent to psychological problems of the burn patients are in a range from 28% to 75% but the symptoms related to physical aspects are 90%.^10^ It has been estimated that the physical performance disorders last up to three years after burn as a result of which muscle strength and volume decline. Physical performance disorders can cause limitations of daily physical activities too.^11^ Severe burns and the influences they have in the form of disability, defect, death and social and economic costs for the society are justified reasons for health care and society’s paying particular attentions to this group of burn victims.^12^ Nowadays, huge, predominantly patient-centered, progresses have been made in health care, treatment and prevention of burns and improvements of survival and quality of life.^13^

The individuals surviving the acute stage of diseases are yet to face numerous physical, psychological and physiological problems that are bitter to their families. Therefore, physical burn injuries are in many respects the worst lesions an individual might experience in acute forms with such chronic symptoms as psychological, social and other disorders stemming from physical performance disorders. Hence, codification of a well-organized, accessible and easy self-care and self-nursing program instigating patient’s cooperation is deemed necessary.^5^^,^^14^ One of the objects of the health care system is improving the performance and enhancing quality of life through devising self-care strategies.^7^

Nurses, as important members of health care system play important role in self-protection of patients and can improve the quality of life of burn patients. Therefore, nursing personnel are needed for taking care of patients and this is quite more accentuated regarding burn patients.^15^ Self-care instruction causes a reduction in the recurrence and clinic visits of burn patients,^7^ but the increase in the self-care knowledge as a means of improvement in quality of life should be provided via instruction.^16^ Patient instruction is an important part of treatment that helps patients participate in self-care systems,^17 ^thereby to empower and keep them bound to the treatment procedures. Moreover, self-care can cause patient satisfaction, readiness for future, disease-related self-efficacy and, on the other hand, enable patients to afford the health care costs through shortening the hospital stays.^18^

As for the implementation of the instructional programs, the selection of a proper instruction method is one of the most significant measures because an effective learning, more than any other method can be provided from a good teaching.^19^ It has been shown that teaching and instruction are the most important principles in transferring and creation of new information and knowledge for the formation of any goal that more audience can be attracted if the instructions are offered based on novel and accessible methods employing media and up-to-date systems. Nowadays, media systems have found a particular position in educational, healthcare and treatment organizations. Meanwhile, virtual teaching has had considerable effects on the self-care instruction of the patients, including CD- and videocassette-based training.^20^

Development of communications and technologies has eased the use of multimedia methods.^21^ Containing text, audio, image and motion pictures, CD-based instruction, as a novel instructional method, provides the transferring of concepts and materials in an easier, more extensive and more fascinating manner.^22^ The studies indicate that the individuals who receive their information via reading can only recall 15% and that the recalling rates can be increased to 25%, if information is received using images and even to 65% when acquired using both images and reading. So images can provide the readers a better way of perceiving and memorizing the information following which the instruction quality is increased.^23^

According to the fact that no intervention study regarding the physical performance aspect of quality of life was found for the burn patients in Iran and also considering the importance of enhancement of quality of life in burn patients and additionally knowing the instructional and research, health care and treatment-protection role of nurses, as mentioned in the above sections, this study assessed the effect of at-dismissal self-treatment CD-based instruction program on the physical performance aspect of burn patients’ quality of life. 

## MATERIALS AND METHODS

The present study was a randomized clinical trial with a control group that was conducted in the wards of Shahid Motahhari Hospital, Tehran, Iran in 2016. The study population included all burn patients hospitalized in this hospital. The inclusion criteria were being in an age range from 18 to 60, having the equipment for using audiovisual of CDs, 10-45 burn percentage, 1^st^, 2^nd^ and 3^rd^ degree burns, minimum literacy of reading and writing and understanding Persian, having no past- sensory and motor problems, no cerebral and psychological disorders, no mental retardation, living in Tehran and its suburbs, having not referred for self-immolation, and not being burnt by electricity. The exclusion criteria were refrainment from continuing with the research and patient death. 

The study sample size was selected based on convenience method and the patients were randomly assigned to intervention and control groups. According to the studies performed on the quality of life and the effect of instruction using a 95% confidence interval and an 80% test power, the number of the required sample size was estimated to be equal to 50 with a consideration of a 10-score difference in the quality of life scores for two groups based on the following formula. It was increased to 55 considering another 10% of dropout likelihood. In the end, 100 individuals were accepted as the study sample. n=2(z1-α/2+z1-β)2s2/(µ1-µ2)2, where, z1-/α2=1.96, z1-β=0.84, s=9 and μ1-μ2=5.

The present study made use of two questionnaires including (i) demographic and disease status questionnaire containing questions about gender, age, job, marital status, burn agent or heat source (benzene, gas, flame, hot liquids, oil, hot food and so forth), educational level, burn degrees, burn percentage and the burnt body part and the questionnaire was completed by the patient and a research colleague on the first day of sample selection and participant’s entry to the research. The questionnaire of quality of life of burn patients (the Burn specific health scale: BSH-B) was designed and used in 2001,^24^ in Iran in 2011^25^ and 2014.^13^ In this study, the BSH-B content validity was reviewed and corrected by 10 university faculty members of medical universities. The reliability was measured through completion of the questionnaire by 20 burn patients admitted in Motahari Hospital and repeating it after 15 days determined by test-retest, Cronbach alpha coefficient of 0.89.

(ii) The burn patient’s quality of life questionnaire contained 40 questions about the rates of skin sensitivities to heat, physical image, hands’ performance, the quality of taking care of burnt regions, communications, abilities to perform simple activities, sexual performance and psychological aspects. The answers were provided in the form of “high”, medium”, “low” and “not at all” and the questions were scored based on a five-point scale from one to five. Based on the questionnaire, quality of life was investigated in each of its aspects separately as well as totally. Out of the 40 questions in questionnaire, 18 were related to physical aspect of quality of life, 11 pertained to psychological aspect of quality of life and the other 11 were connected with social aspect, thereof. The quality of life was calculated based on the obtained mean scores. 

Higher mean scores indicated better quality of life and lower mean scores denoted to low quality of life. A maximum score of 200 revealed a good quality of life. The present study made use of the physical aspect of the questionnaire that was completed by the patient in self-report format on the dismissal day before intervention and three months and six months after intervention. After acquiring the required permits from Iran University of Medical Sciences Ethics Committee, the researchers attended Shahid Motahhari’s Burn Center in Tehran, Iran and explained the study objectives to the hospital’s officials and wards’ supervisors. The patients who were about to be dismissed were randomly selected based on the study inclusion criteria and assigned to control and intervention groups. 

Before the intervention, the demographic questionnaire and burn specifications were completed by the research colleagues using the medical files. Then, the intervention and control groups received face-to-face routine instructions. Plus the routine instructions, self-care instructions specific to the burn patients were additionally provided to the intervention group participants at dismissal within the format of an instructional CD containing text, slide, film and sound. The instructional material were prepared based on the resources pertinent to the training of burn patients (mobility and performing of daily activities and communications, cleaning and sanitation of skin and taking bath and protection of the burn and healed spots and the peeling, skin strip-off, regions, dietary regime, drug use, burn scar pressure garments). At dismissal, each patient was instructed about how to use the CD and answer the questions. 

The quality of life questionnaire was completed once before the intervention and at dismissal by the patient and, in the meantime, the researcher’s telephone number, email address and telegram number were given to the patients, so that they could make calls, if necessary. To perform follow-ups and ascertain the preservation of the study participants, the researcher made phone calls to the intervention and control groups once a week and then, the patients of both of the groups were contacted after three and six months post-intervention and appointments were provided for completing the questionnaire in a self-report form. Finally, the instruction CDs were also provided to the control group in line with observance of research ethics. SPSS software (version 20, Chicago, IL, USA) was used for statistical analysis. Chi-square and Mann-Whitney tests were used to compare the groups. A p value less than 0.05 was statistically considered significant.

## RESULTS

Demographic information of patients in two groups of intervention and control were shown in Table 1. The pre-intervention physical performance of the intervention and control groups were 1.61±0.71 and 1.45±0.47, respectively (*p*=0.41) and were statistically significant until three months post-intervention (*p*=0.001). The mean score of physical performance of the intervention group was higher than that of the control group (*p*=0.001) (Table 2). Moreover, six months post-intervention there was a significant difference between intervention and control groups in terms of physical performance scores, while a higher physical performance score was noticed for intervention group when compared to the control group (*p*=0.001) (Table 3). 

**Table 1:** Demographic information of patients in two groups of intervention and control

**Variable**		**Intervention**		**Control**		**P value**
		**No.**	**%**	**No.**	**%**	
Gender	Female	28	56.00	22	44.00	0.23
	Male	22	44.00	28	56.00	
Age (Year)	18-28	11	22.00	10	20.40	0.35
	29-38	15	30.00	22	44.90	
	39-48	17	34.00	14	28.60	
	49-58	7	14.00	6	6.10	
Marital status	Single	28	56.00	11	20.40	0.001
	Married	22	44.00	39	79.60	
Occupation	Employed	24	48	30	62.50	0.006
	Housewife	12	24	16	33.30	
	Jobless	14	28	4	4.20	
Level of education	Under diploma	2	4.20	4	8.90	
	diploma	26	52.10	33	66.70	
	Bachelor	22	43.80	12	22.20	
	Master of higher	0	0	1	2.20	
Cause of burn injury	Gas	3	6	2	4	0.64
	Natural gas	8	16	14	28	
	Flame	18	36	17	34	
	Liquids	13	26	12	24	
	Kerosine	1	2	2	4	
	Food	4	8	1	2	
	Etc.	3	6	2	4	
Percentage of burn	15-20	12	24	10	20	0.239
	21-26	12	24	18	36	
	27-32	10	20	9	18	
	33-38	8	16	2	4	
	39-45	8	16	11	22	
Degree	1,2,3	30	60	32	64	0.17
	2,3	20	40	18	36	
Area	Hands, Legs	0	0	3	6.3	0.059
	Body, Hands, legs	23	46	13	26	
	Head Shoulder, Hands, Leg	10	20	11	20.8	
	Whole body	17	34	23	47.9	

**Table 2:** Comparison of mean and standard deviation of the physical performance scores obtained for control and intervention groups before the intervention and three months after the intervention

**Group**			**Before intervention**			**Three months after intervention **		
	**No.**	**Mean**	**Standard deviation**	**Test result**	**No.**	**Mean**	**Standard deviation**	**Test result**
Intervention	50	1.61	0.71	Z=-0.82 *p*=0.41	50	3.44	0.95	Z=-6.41 *p*=0.001
Control	50	1.45	0.47		50	2.32	0.37	

**Table 3:** Comparison of mean and standard deviation of the physical performance scores obtained for control and intervention groups after six months post-intervention

**Group**	**No.**	**Mean**	**Standard deviation**	**Test result**
Intervention	50	4.27	0.68	Z=-7.67
Control	50	2.76	0.56	*p*=0.001

**Figure 1:**
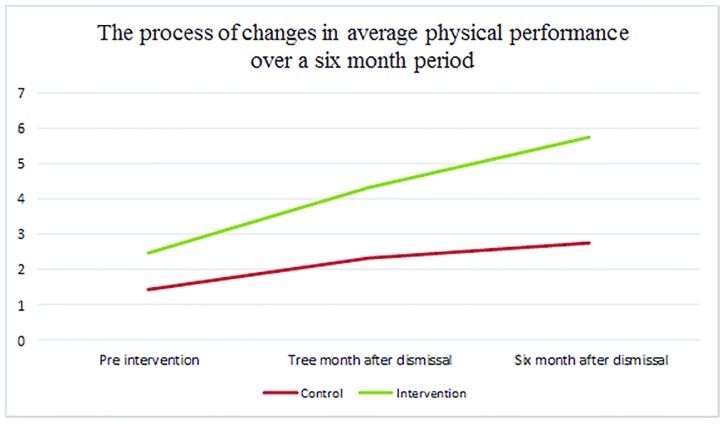
Comparison of the trend of changes in mean physical activity score before, three, and month after intervention

According to Table 4, 17.93, and *p*=0.001, as the significance level was less than 0.05, the assumption indicating the equality of the physical performance score during the three study periods was rejected. So the physical performance score was at least difference in two of the three study periods. Between the two periods, there was a significant difference in terms of physical performance. Dunn follow-up test was employed and the results were presented in Table 5 and, as it can be seen, the mean score of each period was significantly different from the other periods because the significance level was less than 0.05. Figure 1 compares the trend of changes in mean physical activity score before, three, and month after intervention.

**Table 4:** Comparison of mean and standard deviation of the physical performance scores obtained for intervention group before the intervention and at months three and six after the intervention using Friedman test

**Time**	**No.**	**Mean**	**Standard deviation**	**Test result**
Before intervention	50	1.61	0.71	Chi-square=93.17
At month three post-intervention	50	3.44	0.95	DF=2
At month six post-intervention	50	4.27	0.68	*p*=0.001

**Table 5:** Comparison results of intervention group’s physical performance in all three times using Dunn’s pairwise test with Bonferroni’s correction

**Difference**	**Standardized test statistic**	**Corrected significance level**
Before intervention-three months after the intervention	-5.6	Adjusted *p* value=0.001
Before intervention-six months after the intervention	-9.4	Adjusted *p* value=0.001
Three months after the intervention- six months after the intervention	-3.8	Adjusted *p* value=0.001

## DISCUSSION

The present study is indicative of the increase in patients’ physical performance at months three and six following the intervention, hence it can be stated that compact disk-based instructions have resulted in an enhancement in the patients’ physical performance. The same findings have also been mentioned in other studies, as well. In the study by Tang *et al.* (2015), it was shown that the intervention caused an increase in the physical health of burn patients.^2^ Our findings herein are also consistent with what has been reported by Lotfi *et al.* (2018).^10^ In this study undertaken for pre-dismissal intervention’s effects on the quality of life in patients with burns, the control group was provided with routine cares at dismissal and the test group was instructed using questions and responses, instructional pamphlets and a leaflet compiled within the format of a multimedia file. The results indicated that the scores of quality of life was with a significant increases at month three post-intervention.^10^

The study by Elalem *et al.* (2018)^3^ demonstrated that self-care nursing was effective in burn patients and self-care intervention enabled patients to actively take part in their treatment.^3^ Finlay *et al.* performed a study in 2012 with the objective of developing and evaluating DVD-based self-care instructions for the burn out-patients and the findings of the present study are compliant with the aforesaid study, because the improvement in the physical performance scores was reflective of the effectiveness of multimedia self-care instruction.^26^ As it is known, burns are chronic diseases that cause appearance changes, disabilities, difficulties in performing of the occupational duties and familial responsibilities as well as physical, psychological and social outcomes that can substantially influence quality of life. Furthermore, unfavorable quality of life in burn patients causes them to lose independency and become dependent on others.^27^

Daily increasing technological growth as well as the instructions’ becoming virtual in healthcare sector has provided various populations and healthcare providers to face with an important solution parallel to the delivery of healthcare services and it has to be also noted that the development of the instructional infrastructure of healthcare services are of great importance regarding the cost-savings to be followed by.^28^ The daily increase in the advanced technologies provides promising solutions to the problem of having access to the healthcare instruction. The process is in need of the development of newer technologies and cooperation between the society leaders and suppliers of healthcare services.^29^

In the end, considering the studies performed on the effect of self-care instruction based on various methods on the improvement of quality of life and/or on its aspects, it has to be noted that instruction is very effective in patients with chronic diseases and the multimedia compact disk-based instructions are easier, more cost-effective and ubiquitous upon being willing by the patient who can personally refer to it and access the wanted parts in the multimedia disc based on his or her instructional needs. These multimedia compact disks can be used many times till the patients find themselves having reached the required learning threshold without them being in need of any help, hence the patients’ learning needs are readily and easily satisfied. These methods can also be applied in supplementing the other instructional methods. Thus, burn treatment and instruction center’s peer coworkers can use the results of the present study in line with maximal empowering of the burn patients’ instruction. 
